# The sound transmission for clinical diagnosis of developmental dysplasia disease of the hip in newborns: a diagnosis study

**DOI:** 10.3389/fped.2026.1801057

**Published:** 2026-07-27

**Authors:** Nicolás Padilla-Raygoza, Gilberto Flores-Vargas, María de Jesús Gallardo-Luna, Efraín Navarro-Olivos, Luis Guillermo Patiño-Gutiérrez, Ariana Valeria Girón-Soto, Evaristo Antonio Meza-Galván, Itzel Marcela Anguiano-Canchola, Sergio Emmanuel Luna-Santillán, José Juan Torres-Hernández

**Affiliations:** 1 Department of Research and Technological Development, Directorate of Teaching and Research, Institute of Public Health from Guanajuato State, Guanajuato, México; 2 Directorate of Teaching and Research, Institute of Public Health from Guanajuato State, Guanajuato, México; 3 Pediatric Service, Hospital General Guanajuato, Institute of Public Health from Guanajuato State, Guanajuato, México; 4 Ultrasonography Service, Hospital General Guanajuato, Institute of Public Health from Guanajuato State, Guanajuato, México; 5 Surgery Service, Hospital General Guanajuato, Institute of Public Health from Guanajuato State, Guanajuato, México

**Keywords:** developmental dysplasia disease of the hip, luxation, newborns, sound transmission device, subluxation, ultrasonography

## Abstract

**Background:**

Developmental dysplastic hip disease is common in childhood, and clinical maneuvers (e.g., Ortolani and Barlow) detect only subluxation or dislocation. Sound transmission has been proven useful in the diagnosis of this disease.

**Objective:**

to calculate the validity and repeatability of electromagnetic acoustic wave transmitter-receiver device for the clinical diagnosis of hip dysplasia in newborns.

**Design:**

This was a cross-sectional study of newborns at Guanajuato General Hospital whose parents agreed to participate.

**Methods:**

One hundred newborns, between 4 and 27 days of extrauterine life, underwent sound transmission testing with the punctual sound emitter-receiver device placed separately on each hip on three occasions by two examiners. Hip ultrasound was performed using the Graf technique as the gold standard. Validity (sensitivity, specificity, and predictive values) and Cohen's Kappa were calculated to determine the reliability of the device.

**Results:**

Of the 200 hips, 15 right and 18 left hips were diagnosed with dysplasia using the Graf technique ultrasound; a validity greater than 90% was obtained in the three measurements. Repeatability with intra-observer Kappa values were 0.76 for the right hip and 0.82 for the left hip, while inter-observer Kappa values were 0.90 for the right hip and 0.81 for the left hip.

**Conclusion:**

Sound transmission using this clinical device complements the evaluation of the hips of newborns.

## Introduction

Developmental dysplastic disease of the hip (DDDH) is a spectrum of hip abnormalities ranging from mild incongruity between joint surfaces to dislocation of the femoral head outside the acetabulum (1). Causes of DDDH include maternal hormones, macrosomia, breech presentation with extension of the lower extremities, inadequate obstetric practices during birth and postnatal life, and poor swaddling or carrying of the infant (2–4).

DDDH is characterized by physiological immaturity, subluxation, and hip dislocation. Clinical diagnosis is made using maneuvers such as Ortolani, Barlow, Peter Baden, skinfold asymmetry, and limited abduction; however, these maneuvers only detect subluxated and dislocated hips (1–4). Therefore, physiological immaturity may go undiagnosed and become a risk factor for progression to subluxation or dislocation.

Sound transmission with a tuning fork and stethoscope is highly valid and reliable for diagnosing DDDH (5–7); however, examiners may find it difficult to differentiate small variations in bone sound transmission because it is a subjective test. Therefore, an electromagnetic point-to-point acoustic wave transmitter-receiver device (Pat.UGTO) (8) was developed to objectively test sound transmission. The electromagnetic acoustic wave transmitter-receiver device (Pat.UGTO) (8) uses an electromagnetic tuning fork that vibrates at 256 cycles/s, where a receiver is placed to capture the decibels on a log10 scale of sound transmission.

Sound transmission with a tuning fork and stethoscope has shown a sensitivity of 86.36%, which is much higher than the 5.11%, 2.27%, and 5.68% of Ortolani, Barlow, and Peter Baden, respectively, in children (5–7). The electromagnetic acoustic wave transmitter-receiver (Pat.UGTO) (8), which uses sound transmission, has shown a sensitivity of 86.21%, and a Cohen's Kappa of 0.98 (intra-observer) and 0.95 (inter-observer) (9).

Definitive diagnosis in newborns and infants under 4 months of age is made with ultrasound using the Graf technique, which measures alpha and beta angles in static and dynamic tests. Radiographs are not recommended for infants under 8–12 weeks postnatal age owing to the lack of ossification of the femoral head (10, 11).

Delayed diagnosis and treatment of DDDH can result in claudication, hip pain, pelvic tilt, scoliosis, and osteoarthritis. Forty-three percent of end-stage osteoarthritis cases are associated with DDDH (12, 13). Surgical treatment may be required if an infant stands before DDDH correction (13).

As a background to the device used, the Bone Radar (Pat.UGTO) (14) was used in a study involving the hips of 103 newborns (15), and it was applied with the extension/flexion sound transmission test. A sensitivity of 82% and specificity of 96% were reported, similar to that reported in our study of newborns in Guanajuato.

This study aimed to confirm the validity and reliability of an electromagnetic point-to-point acoustic wave transmitter-receiver device (Pat.UGTO) (8) for the clinical diagnosis of DDDH in newborns at the Guanajuato General Hospital (GGH) of the Guanajuato State Public Health Institute (GSPHI).

## Patients and methods

Quantitative cross-sectional diagnostic testing was conducted. The study sample consisted of newborns born at the GGH of the GSPHI in 2024.

Newborns born at the GGH whose parents agreed to participate in the study were selected. The only exclusion criterion was newborns diagnosed with teratological hip dislocation.

No sampling was performed, and all newborns whose parents agreed to participate were included until the sample size reached 100. All newborns between 4 and 27 days old were examined for sound transmission, and hip ultrasonography was performed using the Graf technique. After the parents agreed to participate in the study, the newborn was placed in a supine position with the limbs extended. An electromagnetic tuning fork was placed on one the patella, and a receiver was placed on the pubis. Detailed sound transmission was recorded in log10decibels on the probe screen. The hip was then flexed at 90°. Next, the electromagnetic tuning fork was turned on, and sound transmission in log10decibels was recorded on the screen. Each measurement lasted for 6 s. If the difference in logdecibels was positive between extension/flexion, then the hip had DDDH; if the logdecibels were the same or decreased with the extremity on extension, the hip was considered unaffected (Supplementary video available in supplementary material). The sound transmission test in each hip was performed twice at different times by the same researcher, and a third measure of sound transmission was performed by another researcher. The newborn was scheduled before 28 days of extrauterine life for a hip ultrasound using the Graf technique.

Sociodemographic variables included age at enrollment in the study, sex, gestational age at birth, and mode of delivery.

The difference in log10decibels per hip is a quantitative variable. It was calculated by subtracting the decibels recorded during sound transmission with the lower limb in extension from those recorded with the hip flexed at 90°. It was measured as a positive difference if the measurement with the hip in extension was greater than that with the hip flexed at 90°; it was considered negative if the measurement in extension was less than that in flexion, or if equal to zero. Data are presented as means and standard deviations.

Categorization of the log10decibel difference per hip is a quantitative variable. It was calculated by subtracting the log10decibels recorded during sound transmission with the lower limb in extension from those recorded with the hip flexed at 90°. If the value was negative, the hip was considered affected; however, if the difference was positive, the hip was considered unaffected. The values are presented as mean and standard deviation.

DDDH is a dichotomous categorical variable. It is a hip alteration measured by sound transmission. It was measured as YES if the difference in log10decibels was negative and NO if the difference was zero or positive. The values are presented as frequencies and percentages.

Certainty of developmental dysplastic hip disease is an ordinal categorical variable. It is the hip alteration, measured by ultrasound using the Graf technique through static and dynamic tests, which measures alpha and beta angles. A healthy hip is measured with Graf I (*α* > 60° and *β* < 55°), physiological immaturity is measured with Graf II (*α*: 44°–59° and *β*: 55°–77°), and subluxation or dislocation is measured with Graf III or IV (*α* < 43° and *β* > 77°) (10, 11). The values are presented as frequencies and percentages.

### Sample size

Assuming 90% sensitivity of the device, the minimum sample size calculation is 100 neonates with 95% accuracy and 80% power (EpiIdat, 4.2, Xunta de Galicia, PAHO, WHO, CES University).

### Proposed statistical analysis

Descriptive statistics (frequencies and percentages or mean and standard deviation) were used for all sociodemographic variables. Pearson's chi-square test was calculated, degree of freedom and *P*-value to search for association between sociodemographic variables and risk factors (sex, birth way, family background of DDDH, breech presentation, outcome of first pregnancy, macrosomy, oligohydramnios, and maternal alcoholism) with DDDH for ultrasonography. For validity, the sensitivity, specificity, and predictive values for diagnosing DDDH were calculated using a point-to-point electromagnetic acoustic wave transmitter-receiver device (Pat.UGTO) (8) and hip ultrasound, using the Graf technique as the gold standard. For intra-observer reliability, Cohen's Kappa was calculated using the first and second measurements. However, for inter-observer reliability, Cohen's Kappa was calculated from the first and third measurements.

We examined the association between sociodemographic variables and DDDH diagnosed using ultrasonography. The alpha value was set to.05 in all cases. Statistical analysis was performed using Stata 13.0 (Stata Corp., College Station, TX, USA).

## Results

The 100 enrolled newborns consisted of 56 males; 65% were born vaginally, 5% had a family history of DCS, 4% had a breech presentation, 46% were products of the first gestation, 5% had macrosomia (weight greater than 3,500 g), 9% had oligohydramnios, and only 1% had maternal alcoholism. The mean gestational age was 39.22 ± 1.55 weeks (Table 1). All sociodemographic variables did not show relationship with DDDH by ultrasonography of the hips (Chi-square test with *P* > .05).

**Table 1:** Distribution of sociodemographic variables in newborns from guanajuato (*n* = 100).

Categorical variables		
Variable	n	%
Sex		
Male	56	56.0
Female	44	44.0
Birth way		
Vaginal	65	65.0
Cesarean	35	35.0
Family background of DDDH		
Yes	5	5.0
No	95	95.0
Breech presentation		
Yes	4	4.0
No	96	96.0
Outcome of first pregnancy		
Yes	46	46.0
No	54	54.0
Macrosomy (weight ≥3,500 gr)		
Yes	5	5.0
No	95	95.0
Oligohydramnios		
Yes	9	9.0
No	91	91.0
Maternal alcoholism		
Yes	1	1.0
No	99	99.0
Quantitative variables		
Gestational age (weeks)		
Range	35 to 43.2	
Mean ± SD	39.22 ± 1.55	
Age at ultrasonography (days)		
Range	4 to 27	
Mean ± SD	13.51 ± 7.11	

DDDH, Developmental Dysplastic Disease of the Hip SD standard deviation.

By applying the sound-transmitting device to each hip of the newborns to measure sound transmission, we obtained log10decibel measurements and calculated the difference between extension and flexion; a negative log10decibel difference indicated a hip with dysplasia, and a difference of zero or a positive difference indicated a hip without dysplasia (Table 2).

**Table 2:** Sound transmission and difference in log_10_decibels, by hip from newborns of guanajuato (*n* = 200).

	Measure 1 (log_10_dB)		Measure 2 (log_10_dB)		Measure 3 (log_10_dB)	
	Extension	Flexion	Extension	Flexion	Extension	Flexion
Right hip						
Range	1.8 to 8.1	1.8 to 71	2.2 to 7.5	1.6 to 6.9	1.8 to 7.1	1.3 to 6.3
Mean ± SD	5.01 ± 1.07	4.76 ± 1.11	4.77 ± 1.01	4.59 ± 1.10	4.66 ± 1.11	4.41 ± 1.19
Difference in log_10_decibels						
Range	−1.4 to 3.1		−2.8 to 2.7		−0.7 to 1.6	
Mean ± SD	0.26 ± 0.66		0.36 ± 0.80		0.18 ± 0.34	
Left hip						
Range	2.3 to 7.6	2.1 to 8.4	1.8 to 6.6	1.8 to 7.1	2 to 7.5	1.4 to 7.8
Mean ± SD	5.21 ± 1.07 7	4.83 ± 1.0	4.78 ± 1.04	4.66 ± 1.11	4.78 ± 1.13	4.60 ± 1.18
Difference in log_10_decibels						
Range	−1 to 1.4		−2.4 to 3.1		−2.3 to 2.4	
Mean ± SD	0.12 ± 0.35		0.22 ± 0.54		0.18 ± 0.58	

SD, Standard deviation.

Hip ultrasonography using the Graf technique is the gold standard. Three measurements of the right and left hips with the electromagnetic transmitter-receiver showed 1–4 false positives in the right hip and 1–5 false positives in the right hip; similarly, the false negatives ranged from 1 to 4 in the right hip and 1–5 in the left hip (Table 3).

**Table 3:** Distribution of diagnosis of dysplasia of the hip by sound transmission and ultrasonography of hips with graff's technique (*n* = 200).

	Right hip (*n* = 100)		Left hip (*n* = 100)	
	Dysplasia Non dysplasia		Dysplasia Non-dysplasia	
	n	n	n	n
First measure				
Sound transmission	19	81	19	81
Second measure				
Sound transmission	16	84	23	77
Third measure				
Sound transmission	16	84	21	79
Ultrasonography Graff's technique	15	85	18	82

Hip ultrasonography alone using the Graf technique revealed 13 cases of right hip instability, 1 case of subluxation, 1 case of dislocation, 17 cases of left hip instability, and 1 case of dislocation (Table 4).

**Table 4:** Diagnosis of hip with graf technique ultrasonography.

	Ultrasonography right hip		Ultrasonography left hip	
	*n*	%	*n*	%
Graf I	85	85.00	82	82.00
Graf II	13	13.00	17	17.00
Graf III	1	1.00	0	0.00
Graf IV	1	1.00	1	1.00

The validity of the sound transmitter-receiver device was very consistent across the three measurements of both the right and left hips, achieving a validity greater than 90%, with sensitivity ranging from 83.33% to 93.33%, sensitivity ranging from 90.36% to 97.65%, positive predictive values ranging from 69.57% to 87.50%, and negative values ranging from 96.20% to 98.77% (Table 5).

**Table 5:** Distribution of the diagnosis of dysplasia of the hip and validity of sound transmission with gold standard (ultrasonography with graf's technique) (*n* = 100).

Diagnosis on sound transmission	US hip right		US hip left		Validity	Sensitivity	Specificity	+ PV	−PV
	Dyspl Non-dyspl		Dyspl Non-dyspl		%	%	%	%	%
First measurement									
Hip right					94.00	93.33	94.12	73.68	98.77
Dysplasia	14	5							
Non-dysplasia	1	80							
Hip left					90.00	83.33	94.94	78.94	96.30
Dysplasia			15	4					
Non-dysplasia			3	78					
2° measurement									
Hip right					93.00	80.00	95.29	75.00	96.43
Dysplasia	12	4							
Non-dysplasia	3	81							
Hip left					91.00	88.89	90.36	69.57	97.40
Dysplasia			16	7					
Non-dysplasia			2	75					
Third measurement									
Hip right					97.00	93.33	97.65	87.50	98.81
Dysplasia	14	2							
Non-dysplasia	1	83							
Hip left					91.00	83.33	92.68	71.43	96.20
Dysplasia			15	6					
Non-dysplasia			3	76					

US Ultrasonography Dyspl Dysplasia of the hip Non-dyspl No dysplasia of the hip +PV, Positive predictive value; −PV Negative predictive value.

Table 6 shows the reliability results; Cohen's Kappa values were 0.76 for the right hip and 0.82 for the left hip. When the first and second measurements were compared (performed by the same researcher), the Kappa value was 0.90 for the right hip, and when the first and third measurements were compared (performed by different researchers), the Kappa value was 0.81 for the left hip.

**Table 6:** Repeatability of sound transmission with Cohen's kappa (*n* = 200).

	Second measurement				Third measurement			
	Hip right		Hip left		Hip right		Hip left	
	Dyspl	Non-dyspl	Dyspl	Non-dyspl	Dyspl	Non-dyspl	Dyspl	Non-dyspl
First measure								
Hip right								
Dysplasia	14	5			16	3		
Non-dysplasia	2	79			0	81		
Hip left								
Dysplasia			18	1			17	2
Non-dysplasia			5	76			4	77
Cohen's Kappa	0.76		0.82		0.90		0.81	
CI 95%	0.59–0.93		0.68–0.96		0.78–1.00		0.67–0.96	

Dyspl Dysplasia of the hip Non-dyspl No dysplasia of the hip.

## Discussion

The study sample comprised hips of 100 newborns born at the GGH. Of these, 56% were male, 64% were delivered vaginally, and the gestational age was 39.22 ± 1.55 weeks (Table 1). In a previous study involving the hips of 100 newborns in Celaya, Mexico, Padilla et al. (9) reported a predominance of females (64%) and detected dysplasia in 29 (14.5%) hips. In the sample of newborns from GGH, the frequency of DDDH was 16.5% (Table 4).

Singh et al (16), reported the sensitivity of sound transmission for DDDH in 80% and specificity 50%–79%, results similar that those obtained in newborns from Guanajuato, Mexico.

In this study, we found no relationship between risk factors and DDDH (*P* > .05) (Table 1), which differs from the report by Ömeroglu et al. (17).

Using hip ultrasound with the Graf technique as the gold standard and sound transmission with the device as a new test, the sensitivity at the first measurement was 87.88%, and the positive predictive value was 76.32%. With the second sound transmission measurement, the sensitivity was 84.85% and the positive predictive value was 71.79%; the third measurement yielded a sensitivity of 87.88% and a positive predictive value of 78.38% (Table 2). Padilla et al. (9) reported sensitivity, specificity, and predictive values similar to those of the first prototype of the sound transmitter-receiver device.

Using the tuning fork, Stone et al. (5), and Padilla et al. (4, 7) reported sensitivity and specificity values higher than 70%, but the sound transmission tests with a tuning fork and stethoscope are subjective tests. However, the sound transmitter-receiver device allows for an objective evaluation of the hips of newborns.

### Strengths

The use of sound transmission for clinical diagnosis, whether with a tuning fork and stethoscope (4–7), bone radar (Pat.UGTO) (14), or the electromagnetic transmitter-receiver device (Pat.UGTO) (8, 9), demonstrates good validity and reliability, allowing for improved diagnosis and monitoring of newborns with dysplasia.

### Weaknesses

The sample size of newborns was small. Further studies with larger sample sizes as well as studies with follow-up of newborns until 1–2 years old are needed to demonstrate the benefits of sound transmission.

Our search in electronic scientific databases revealed that only Stone et al. (5) and Padilla et al. (4, 6, 7, 9), and Singh et al. (16) have worked on sound transmission for the clinical diagnosis of DDDH.

## Conclusion

Sound transmission complements to the maneuvers for the clinical diagnosis of DDDH; it does not substitute ultrasonography of the hips. Graf technique is the gold standard for the diagnosis of DDDH.

## Data Availability

The datasets presented in this study can be found in online repositories. The names of the repository/repositories and accession number(s) can be found in the article/Supplementary Material.

## Supplementary material

Supplementary Video The newborn is placed supine with the lower extremities extended. The device is turned on, and the electroacoustic vibrator (green) is placed over the patella and the receiver (stethoscope) over the pubis. The device transmits sound for approximately 6 seconds, and the receiver sends the log10 decibel signal to the screen. The hip is flexed, and the electroacoustic vibrator is placed over the patella and the receiver over the pubis. The receiver transmits the sound to the screen in log10 decibels. If the sound is greater than flexion, the hip is dysplastic; if the sound is equal to or less than flexion, the hip is normal.
